# Current Options and Future Directions for NAFLD and NASH Treatment

**DOI:** 10.3390/ijms22147571

**Published:** 2021-07-15

**Authors:** Chunye Zhang, Ming Yang

**Affiliations:** 1 Department of Veterinary Pathobiology, University of Missouri, Columbia, MO 65211, USA; czvw9@mail.missouri.edu; 2 Department of Surgery, University of Missouri, Columbia, MO 65211, USA

**Keywords:** nonalcoholic fatty liver disease, nonalcoholic steatohepatitis, molecules, signaling pathway, treatment options, clinical trials

## Abstract

Nonalcoholic fatty liver disease (NAFLD) is the most common chronic liver disease worldwide, with a broad spectrum ranging from simple steatosis to advanced stage of nonalcoholic steatohepatitis (NASH). Although there are many undergoing clinical trials for NAFLD treatment, there is no currently approved treatment. NAFLD accounts as a major causing factor for the development of hepatocellular carcinoma (HCC), and its incidence rises accompanying the prevalence of obesity and diabetes. Reprogramming of antidiabetic and anti-obesity medicine is a major treatment option for NAFLD and NASH. Liver inflammation and cellular death, with or without fibrosis account for the progression of NAFLD to NASH. Therefore, molecules and signaling pathways involved in hepatic inflammation, fibrosis, and cell death are critically important targets for the therapy of NAFLD and NASH. In addition, the avoidance of aberrant infiltration of inflammatory cytokines by treating with *CCR* antagonists also provides a therapeutic option. Currently, there is an increasing number of pre-clinical and clinical trials undergoing to evaluate the effects of antidiabetic and anti-obesity drugs, antibiotics, pan-caspase inhibitors, *CCR2*/*5* antagonists, and others on NAFLD, NASH, and liver fibrosis. Non-invasive serum diagnostic markers are developed for fulfilling the need of diagnostic testing in a large amount of NAFLD cases. Overall, a better understanding of the underlying mechanism of the pathogenesis of NAFLD is helpful to choose an optimized treatment.

## 1. Introduction

Nonalcoholic fatty liver disease (NAFLD) is the most common chronic liver disease worldwide, ranging from simple hepatic steatosis to advanced stage of nonalcoholic steatohepatitis (NASH), which may lead to liver fibrosis, cirrhosis, and hepatocellular carcinoma (HCC) [1]. Recently, a new definition was suggested for NAFLD, namely metabolic dysfunction-associated fatty liver disease (MAFLD) [2]. The prevalence of MAFLD among obese adults worldwide is 50.7%, with 95% CI 46.9–54.4 [3], relatively higher in men (59.0%, 95% CI 52.0–65.6) than women (47.5%, 95% CI 40.7–54.5). NAFLD is tightly associated with obesity, diabetes, and metabolic syndromes [4,5]. A survey study showed that over the past three decades, NAFLD is the only consistently increasing liver disease in the United States, accompanying the increase in obesity and type 2 diabetes mellitus (T2DM) [5]. In addition to Western countries, the prevalence of NAFLD also increases in the past two decades in Asian countries, due to the sedentary lifestyle, overnutrition, obesity, and T2DM [6].

Both genetic and epigenetic factors impact the development and progression of NAFLD and NASH [7,8]. For example, the allele variant of rs738409 C > G in the patatin-like phospholipase domain containing 3 (*PNPLA3*) can increase the susceptibility of NAFLD and NASH, which was found from studies in Brazil [9,10]. In addition, NASH patients with *PNPLA3* GG alleles had a higher level of aspartate aminotransferase (AST) and advanced liver fibrosis compared to patients with *PNPLA3* CC alleles [10]. As the most evaluated epigenetic factor, DNA methylation in the CpG islands can be applied to test the progression of NAFLD to liver fibrosis and HCC [11,12]. These factors have been well described in other review papers [13,14], which will not be discussed here. 

In the past decade, non-invasive diagnostic techniques and novel therapeutic strategies have been developed. In this review, we first discuss the underlying molecular mechanisms of the pathogenesis of NAFLD and NASH. Then, we summarize the latest progression of diagnostic markers applied in NAFLD, NASH, or both. Following the investigation of the molecules in the pathogenesis and diagnosis of NAFLD and NASH, the potential therapeutic targets are summarized. Finally, treatment options in pre-clinical trials and clinical trials are discussed.

## 2. Important Molecules and Their Mediated Signaling Pathways in NAFLD and NASH

**Figure 1:**
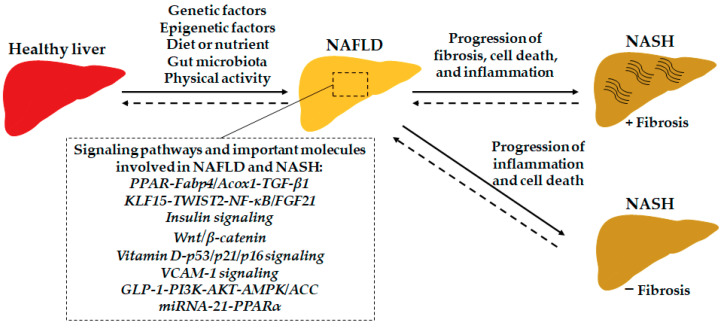
The important molecules and signaling pathways are involved in the development of NAFLD and the progression of NASH. Abbreviations: *ACC*, acetyl-CoA carboxylase; *Acox1*, acyl-CoA oxidase 1; *AKT*, protein kinase B; *AMPK*, AMP-activated protein kinase; *Fabp4*, fatty acid-binding protein 4; *FGF21*, fibroblast growth factor 21; *KLF15*, Krüppel-like factors 15; *GLP-1*, glucagon-like peptide-1; *NF-κB*, nuclear factor (NF)-κB; *PI3K*, phosphoinositide 3-kinase; *PPAR*, peroxisome proliferator-activated receptor; *TWIST2*, twist-related protein 2; *VCAM-1*, vascular cell adhesion molecule 1.

There are some essential molecules and their associated signaling pathways involved in the progression of NAFLD by mediating the lipid and sugar metabolism, cell apoptosis, liver inflammation and fibrosis, and so on. For example, the well-known transforming growth factor-beta 1 (*TGF-β1*) signaling pathway plays critical roles in liver cell apoptosis [15], inflammation [16], NAFLD, and NAFLD-related HCC progression [17,18]. The signaling pathways involved by important molecules are potential targets for developing NAFLD and NASH diagnosis and treatment. Here, we summarize some latest findings in this field (Figure 1). 

### 2.1. Peroxisome Proliferator-Activated Receptors

The peroxisome proliferator-activated receptors (*PPARs*) are nuclear receptor proteins and play a vital role in modulating fatty acid and glucose metabolism. There are three subtypes of *PPARs*: *PPARα*, *PPARβ*/*δ*, and *PPARγ*. Currently, the single and dual *PPAR* agonists have been applied in the clinic for the treatment of hyperlipidemia, T2DM, metabolic syndrome and associated cardiovascular diseases [19]. For example, a *PPARγ* activator, rosiglitazone approved by the FDA for the treatment of T2DM, showed effects against steatosis, hepatocellular inflammation, ballooning degeneration, and fibrosis [20]. A clinical trial study showed that treatment with lobeglitazone, a dual *PPARα/δ* agonist, at a dose of 0.5 mg daily for 24 weeks, significantly improved glycemic, hepatic steatosis, and serum enzymes for liver damage in T2DM patients with NAFLD [21]. Another antidiabetic drug elafibranor (GFT505), a dual *PPARα/δ* agonist, can improve NASH and multiple cardiometabolic risk factors associated with metabolic syndrome and T2DM without worsening fibrosis at a dose of 120 mg/d for one year [22]. Molecular mechanism study showed that elafibranor can promote lipid metabolism by upregulating genes such as fatty acid-binding protein 4 (*Fabp4*) and acyl-CoA oxidase 1 (*Acox1*), and inhibit liver inflammation and liver fibrosis, evidenced by inhibited expression of genes such as C-C motif chemokine ligand 6 (*CCL6*), *TGF-β1*, type one collagen alpha 1 (*Col-1A1*), and tissue inhibitor of metalloproteinases 1 (*TIMP-1*) [23].

### 2.2. Krüppel-Like Factors

Krüppel-like factors (*KLFs*) are transcription factors that play pivotal roles in diseases. For example, *KLF10*-deficient mice on a high-sucrose diet promoted the progression of hepatic steatosis, inflammation, and fibrosis compared to wild-type mice [24]. Another study showed that *KLF15* can activate twist-related protein 2 (*TWIST2*) to ameliorate liver steatosis and inflammation by modulating nuclear factor (*NF*)*-κB* or sterol regulatory element-binding protein 1c (*SREBP1c*)-fibroblast growth factor 21 (*FGF21*) signaling pathways [25]. Global knockdown of *KLF6* or hepatocyte-specifical knockdown of *KLF6* can improve glucose and lipid metabolism, as well as insulin tolerance by attenuating the function of *PPARα* [26]. However, most of the current studies are pre-clinical investigations, and so the significant role of *KLFs* in human liver pathogenesis remains to be clarified. 

### 2.3. Insulin Signaling Pathway

Insulin resistance (IR) is a feature of metabolic dysfunction, which contributes to the development of NAFLD and NASH. Hepatic insulin resistance can induce dyslipidemia and increase the development of atherosclerosis, as 100% of hepatic insulin receptor knockout (LIRKO) mice developed hypercholesterolemia but not wild-type mice on an atherogenic diet for 12 weeks [27]. An increase in IR can impair glucose homeostasis in NAFLD patients [28]. Treatment of Eugenol, an aromatic oil extracted from cloves, activated insulin receptor substrate-2 (*IRS-2*) to improve IR evidenced by reducing Homeostasis model assessment for insulin resistance (HOMA-IR) and hepatic triglycerides [29]. Improving hepatic insulin sensitivity by treatment of insulin sensitizers can also improve glycemia or glycose tolerance and liver function [30]. Treatment with pioglitazone, an antidiabetic drug for T2DM, can enhance insulin signaling and increase glucose uptake and lipid metabolism in adipose tissue and reduce liver gluconeogenesis [31]. In addition, pioglitazone shows a therapeutic effect against NAFLD/NASH by reducing hepatic steatosis and anti-lobular inflammation via regulating *PPARγ* signaling and mitochondrial gene expression [32,33]. 

### 2.4. Wnt Signaling Pathway

The *Wnt* family consists of 19 members in human. Among them, *Wnt1*, *Wnt2*, *Wnt2b*, *Wnt3*, *Wnt3a*, *Wnt7a*, *Wnt8*, *Wnt8b*, and *Wnt10a* are involved in the canonical *Wnt* signaling pathway, while *Wnt4*, *Wnt5a*, and *Wnt11* are implicated in the noncanonical *Wnt* signaling pathway [34]. Activation of canonical *Wnt*/*β*-catenin signaling pathway can enhance the development and progression of primary liver cancers [35,36]. Canonical *Wnt*/*β*-catenin and non-canonical *Wnt* signaling pathways are also implicated in the progression of NASH and liver fibrosis. For example, the expression of aortic carboxypeptidase-like protein (*ACLP*), a secreted glycosylated protein in hepatic stellate cells (HSCs), is associated with mouse and human NASH by activating canonical *Wnt*/*β*-catenin [37]. The expression of *Wnt5a* and *Wnt11* was increased by 3-fold and 15-fold, respectively, in the diet-induced mouse NASH model, indicating the involvement of non-canonical *Wnt* signaling in NASH progression [38]. 

### 2.5. p53 Signaling Pathway

Tumor suppressor gene *p53* plays an important role in the pathogenesis of NAFLD and NASH [39,40]. For example, silencing *p53* in human liver cancer cell lines HepG2 cells and Huh7 cells decreased palmitate-induced lipid accumulation [41]. In addition, *p53*-deficient mice significantly reduced hepatic lipid accumulation and high-fat diet (HFD)-induced NAFLD symptoms in vivo. Supplementation of vitamin D can reduce the senescence and apoptosis of hepatocytes via inhibiting *p53*, *p21*, and *p16* signaling pathways, resulting in amelioration of NAFLD [42]. In contrast, long-term activation of *p53* with low-dose of doxorubicin showed a beneficial effect on the HFD-induced murine NAFLD model, including reduction of lipogenesis, inflammation, and endoplasmic reticulum (ER) stress [43], which was abrogated in liver-specific *p53* deficiency mice. Moreover, *p53* is also implicated in the pathogenesis of liver fibrosis [44,45] and HCC [46,47].

### 2.6. Vascular Cell Adhesion Molecule 1

Liver sinusoidal endothelial cells (LSECs), hepatic gatekeeper cells, play critical roles in liver diseases as discussed in our previous publication, including NAFLD, liver fibrosis, cirrhosis, and HCC [48]. Hepatic infiltration of inflammatory monocytes and lymphocytes is of critical importance in liver injury. Under the lipotoxic condition, the expression of vascular cell adhesion molecule 1 (*VCAM-1*) on LSECs is significantly increased in murine and human NASH [49], which mediates the migration of inflammatory cells and results in the progression of NASH [50]. In addition, the serum level of *VCAM-1* is an independent marker to predict advanced liver fibrosis (≥F2) in NAFLD patients, showing a sensitivity of 80% and specificity of 83% at the cutoff point of 13.2 ng/mL [51].

### 2.7. Glucagon-Like Peptide-1

Glucagon-like peptide-1 (*GLP-1*) receptor agonists have been approved for the treatment of type 2 diabetes and obesity. Intraperitoneal treatment of a synthetic peptide AWRK6, a candidate agonist of *GLP-1*, can improve hepatic steatosis, glucose metabolism, and obesity in high energy diet (HED)-induced MAFLD mice, via modulating phosphoinositide 3-kinase (*PI3K*)/Protein kinase B (*AKT*)/AMP-activated protein kinase *(AMPK)*/acetyl-CoA carboxylase *(ACC)* signaling pathway [52]. In diabetic fatty rats, *GLP-1* agonists liraglutide and Ex-4 can enhance the expression of *PPARα* through a *GLP-1* receptor/*AMPK* signaling pathway [53]. However, a cohort study showed that the treatment of *GLP-1* agonist did not decrease the risk of NAFLD development compared to insulin treatment [54]. Therefore, more studies are needed to clarify the therapeutic role of *GLP-1* in NAFLD.

### 2.8. MicroRNAs

The microRNAs (miRNAs) of epigenetic factors play essential roles in each step of the development and progression of NAFLD. For example, liver-specific *miR-21* depletion can inhibit obesogenic diet-induced steatosis and glucose intolerance in mice [55]. Inhibiting *miR-21* expression can also suppress a methionine-choline-deficient (MCD) diet-induced liver inflammation and fibrosis by recovering the function of *PPARα*, evidenced by loss of function of inhibitor of *miR-21* in *PPARα*-deficient mice [56]. Different miRNAs can modulate different molecules or signaling pathways to impact the progression of NAFLD and NASH, which are summarized in Table 1.

## 3. Serum Marker for the Diagnosis of NAFLD and NASH 

NAFLD is a broad spectrum of liver disease, ranging from early steatosis to NASH with advanced liver inflammation and fibrosis. The basis of treatment is dependent on the stage of NAFLD. Therefore, early diagnosis of NAFLD and the advanced stage of NASH is critically important for selecting appropriate treatment. Currently, liver biopsy is the gold standard for NAFLD diagnosis [66]. However, it is invasive and expensive and may cause improper diagnosis due to sampling bias [1]. Multi-omics have been used to investigate new non-invasive markers for the diagnosis of NAFLD and advanced liver disease [67,68]. A significant reduction of hepatic fat with advanced liver fibrosis in patients with NASH, even to the point of complete fat loss, burnt-out NASH [69]. An increase in serum adiponectin level was significantly associated with burnt-out NASH [70]. Here, we summarize the serum markers and score system for detecting NAFLD and NASH (Table 2). Performing the analysis of the area under the receiver operating characteristic curve (AUROC) is commonly applied to evaluate the applicability of models or score systems. 

## 4. Treatment Options for NAFLD and NASH 

The liver is an essential organ for energy metabolism, and dysfunction of energy metabolism or metabolic syndrome impacts its function, resulting in the progression of NAFLD and NASH. Therefore, strategies modulating the change of metabolic dysfunction can be applied to treat liver diseases.

### 4.1. Lifestyle Modification

Lifestyle modification is an effective way of prevention and treatment of NAFLD. The main purpose of lifestyle modification, such as a healthy diet and exercise, is to keep appropriate body weight. 

#### 4.1.1. Calorie-Restricted Diet or Low-Fat Diet

Consumption of a diet with calories less than the required daily energy, such as the Mediterranean diet, can reduce body weight, hepatic lipid accumulation, and insulin resistance, as well as decreased serum levels of saturated fatty acid and increased serum levels of monounsaturated and n-3 polyunsaturated fatty acid [87]. In a controlled clinical trial, 74 patients with NAFLD were randomized in a 1:1:1 ratio to a 12-week treatment with either a 5:2 diet with an intermittent calorie restriction (500 kcal/day for women and 600 kcal/day for men) for two non-consecutive days per week, or a low-carbohydrate high-fat diet (LCHF) with an average daily calorie intake of 1600 kcal/day for women and 1900 kcal/day for men, or general lifestyle advice from a hepatologist by choosing a healthy diet, doing exercise, reducing alcohol, and others [88]. The results indicated that both the 5:2 diet and LCHF are more effective to reduce hepatic steatosis and body weight compared to general lifestyle modification.

Dietary intervention can also modulate the components of gut microbiota and improve the health condition of NAFLD patients [89], such as reduction of body weight and improvement of insulin resistance. Consumption of a Dietary Approaches to Stop Hypertension (DASH) diet can reduce BMI, serum markers of alanine aminotransferase (ALT), alkaline phosphatase (ALP), insulin levels, and HOMA-IR compared to the control diet [90]. 

#### 4.1.2. Exercise

A cross-sectional study in Korea showed that adults who have long working hours (53–83 h/week) are more likely to develop NAFLD compared to those who work the standard hours (36–42 h/week), especially in women workers and workers with age < 63 years [91]. This result was concluded after adjusting factors including age, sex, BMI, smoking, alcohol, exercise, diabetes, hypertension, and serum triglyceride and total cholesterol [91]. Another study in Japan showed that there was a significant association between working hours and metabolic syndrome in men workers age ≥ 40 years [92]. The workers who worked 8–9 h/day had an odds ratio of 2.02 (95% CI, 1.04–3.90) and those working > 10 h/day with an odds ratio of 3.14 (95% CI, 1.24–7.95) in developing metabolic syndrome compared with those who worked 7–8 h/day [92]. In addition, long hours compared to standard hours may increase the risk of other diseases, such as coronary heart disease [93] and stroke [94]. However, when interpreting clinical trial results or design clinical trials for lifestyle modification, introducing some uncertainties such as Hawthorne effects among participants may result in alteration of their behavior and trial results [95,96]. Therefore, appropriate controls are critically important in these kinds of trials.

Long-term exercise can prevent NASH development by improving the phagocytic capacity of liver resident Kupffer cells (KCs) and reducing liver inflammation and fibrogenesis [97]. A murine study showed that maternal exercise can reduce western-style-diet (WSD)-induced obesity and improve hepatic lipid metabolism via activating the AMP-activated protein kinase (*AMPK*) and *PPARγ*-coactivator-1α (*PGC-1α*) signaling pathways [98]. Exercise-training intervention can also reduce intrahepatic fat accumulation, blood pressure, and insulin resistance in obese adults [99]. However, the working pressure and fast-food products make the change of lifestyle very difficult or cause most people to give up in this process. 

### 4.2. Bariatric Surgery

Bariatric surgery (BS) or weight loss surgery is considered the most effective way to treat obesity and diabetes [100,101], by reducing food absorption and modulating gut hormone secretion and metabolic dysfunction. A meta-analysis showed that BS can significantly reduce mortality and expand lifetime in adults with obesity compared to usual obesity management [102]. The bariatric procedures sleeve gastrectomy (SG) and Roux-en-Y gastric bypass (RYGB) on NAFLD and NASH are similarly effective, evidenced by the change of liver function tests (LFTs) after one year of surgery [103]. Other studies indicate that there are some differences induced by BS procedures [104,105]. For example, a 5-year follow-up study (a long-term effect) showed that laparoscopic Roux-en-Y gastric bypass (LRYGB) was better to improve weight loss and reduce hypertension compared to laparoscopic sleeve gastrectomy (LSG), but there was no significant difference in remission of T2DM, obstructive sleep apnoea, or improvement in quality of life, data collected from two randomized clinical trials [105]. In patients aged ≥ 65, the effect of LRYGB on controlling weight loss, HbA1c (diabetes), and low-density lipoprotein (LDL) is better than LSG [106].

### 4.3. Modification of Gut Microbiota

Dysbiosis of gut microbiota is a causing factor for NAFLD, due to the change of gut hormones, metabolites, inflammatory factors. The abundance of some bacterial species is associated with the progression of liver disease. For example, there is a negative correlation between the severity of liver fibrosis and *Eubacterium* abundance in non-obese patients with NAFLD [107]. The abundance of *Ruminococcaceae* and *Veillonellaceae* is also positively associated with the severity of liver fibrosis in non-obese subjects [108]. Modulation of gut microbiota via different strategies [109], including fecal microbiota transplantation (FMT), drug therapy (e.g., antibiotics), modification of lifestyle (e.g., dietary change), the above-mentioned BS, and others can improve liver disease. A meta-analysis study showed that supplementation of probiotics can reduce the expression of inflammatory factors, such as tumor necrosis factor-α (*TNF-α*) and C-reactive protein (*CRP*) in the livers of NAFLD patients [110]. Allogenic fecal microbiota transplantation (FMT) from thin and healthy people to patients with NAFLD can reduce the small intestinal permeability post-six-week treatment [111]. However, the effect of FMT on liver dysfunction and metabolic syndrome remains minimal. A double-blind phase 2 trial also showed that a yearly treatment of synbiotics, a combination of probiotic and prebiotic, only modulated the change of gut microbiota but not the liver fat content and fibrotic markers [112]. 

Furthermore, BS modulates the components of the gut microbiota profile. For example, the predominant bacteria of *Bacteroides* before surgery was decreased after laparoscopic gastric bypass (LGB) [113]. Both the altered bacterial profile and their associated metabolism following BS contribute to the impact on patients [114]. 

### 4.4. Medicines

#### 4.4.1. Antidiabetic and Anti-Obesity Drugs

The incidence of NAFLD is significantly associated with T2DM and obesity, especially in patients with a higher body mass index (BMI) [115,116]. In contrast, the incidence of NAFLD is decreased in patients with T2DM, who received treatments such as sodium-glucose cotransporter-2 (*SGLT2*) inhibitors, *GLP-1* receptor antagonists, and insulin [115]. The reprogramming of antidiabetic or anti-obesity drugs, such as pioglitazone and saroglitazar, are being considered as the options for NAFLD/NASH treatment. Saroglitazar, a double agonist against *PPAR-α/γ*, can reduce hepatic lipid accumulation, lobular inflammation, hepatocyte ballooning, and liver fibrosis in a mouse NASH model [117]. A phase 2 clinical trial study showed that 4 mg of saroglitazar can significantly decrease ALT and liver fat content, improve insulin resistance and dyslipidemia in patients with NAFLD or NASH [118].

Farnesoid X receptor (*FXR*), a nuclear receptor that can be activated by bile acids (BAs), plays a critical role in hepatic lipid accumulation [119,120,121], as well as glucose homeostasis [122]. Cilofexor (GS-9674), a nonsteroidal agonist of *FXR* with an EC_50_ of 43 nM, has anti-inflammatory and antifibrotic effects. A phase 2 trial study showed that cilofexor significantly improved hepatic steatosis and decreased serum γ-glutamyltransferase, C4, and primary bile acids, but not liver fibrosis and stiffness in NASH patients [123].

*SGLT2* inhibitors, such as canagliflozin, dapagliflozin, and empagliflozin, have pleiotropic functions to treat NAFLD and T2DM by preventing de novo lipogenesis, liver inflammation and cell apoptosis, and increasing fatty acid oxidation [124]. 

*GLP-1* receptor agonists can inhibit hepatic fat accumulation as the effect of metformin and insulin-based treatment [125]. In addition, they can moderately improve liver fibrosis. Metformin is the first-line treatment for patients with T2DM, which can control high blood sugar [126] and decrease total cholesterol, LDL, and triglycerides [127]. However, the effects of metformin, as well as dipeptidyl peptidase-4 (*DPP-4*) inhibitors in NAFLD treatment, remain debatable [128]. 

#### 4.4.2. Antioxidants

A meta-analysis of clinical trials showed that adjuvant vitamin E treatment is favorable for adult patients with NAFLD compared to pediatric patients [129]. Vitamin E can also improve NASH in HIV-infected patients, evidenced by the reduction of serum biomarker ALT and cytokeratin 18 (*CK-18*) for hepatocyte apoptosis [130]. 

Polyphenols as anti-inflammatory and antioxidant reagents show a protective effect in liver disease, and consumption of polyphenol-rich diets also pride beneficial effects for NAFLD patients [131]. For example, a high intake of lignans, a large group of low molecular weight polyphenols in plants such as whole grains, reduces the incidence of NAFLD [132].

#### 4.4.3. Antibiotics

Rifaximin has been used for treating hepatic encephalopathy, and it also has a protective effect against intestinal leaking [133]. A 28-day treatment of rifaximin (1200 mg/day) in NASH patients resulted in a dramatic reduction of serum endotoxin, AST, ALT, γ-glutamyltransferase, LDL, and ferritin, and a mild reduction of the average BMI [134]. However, the effect of rifaximin on patients with simple steatosis was significantly reduced, with only effects on the reduction of ALT and ferritin [134]. 

#### 4.4.4. Anti-Cell Death Reagents

Lipotoxicity causes hepatic cell death and promotes the progression of NAFLD and NASH, which induces proinflammatory cytokines and chemokines and activation of hepatic stellate cells (HSCs). Therefore, inhibiting cellular death is critically important for the treatment of chronic liver disease. For example, selonsertib, a selective inhibitor of apoptosis signal-regulating kinase 1, showed an antifibrotic effect in patients with NASH and F2-F3 liver fibrosis [135]. However, two phase 3 clinical trials showed that selonsertib monotherapy did not improve liver fibrosis in patients with NASH and bridging fibrosis (F3) or compensated cirrhosis (F4) compared to placebo control treatment [136]. In another phase 2 trial showed that a combination of selonsertib with cilofexor or firsocostat (a small molecule inhibitor of acetyl-CoA carboxylase) improved steatosis, while a combination of selonsertib with cilofexor improved the early stage of liver fibrosis (F1) [137]. These completed trials showed that selonsertib has some effects on the early stage of NASH, but not advanced liver fibrosis or cirrhosis. Metformin, a first-line antidiabetic drug, also shows a protective effect against palmitate-induced necrosis in primary rat hepatocytes by reducing reactive oxygen species (ROS) and improving mitochondrial function [138]. 

#### 4.4.5. Antifibrotic Reagents

Antifibrotic reagents can prevent the progression of liver fibrosis and NAFLD to fibrotic NASH. The activated HSCs are a source of predominant extracellular matrix proteins-producing cells during liver fibrosis [139,140]. Anti-liver fibrosis methods mainly consist of inhibiting the activation and growth of HSCs, anti-inflammation, anti-cell death agents, and regulating the production of extracellular matrix (*ECM*) proteins. The abovementioned methods for the treatment of NAFLD or NASH are also treatment options for liver fibrosis. Some natural products have multiple effects. For example, Scoparone, a bioactive compound from a Chinese herb, can decrease hepatic steatosis, inflammation, cell death, and fibrosis in mice with diet-induced NASH [141].

**Figure 2:**
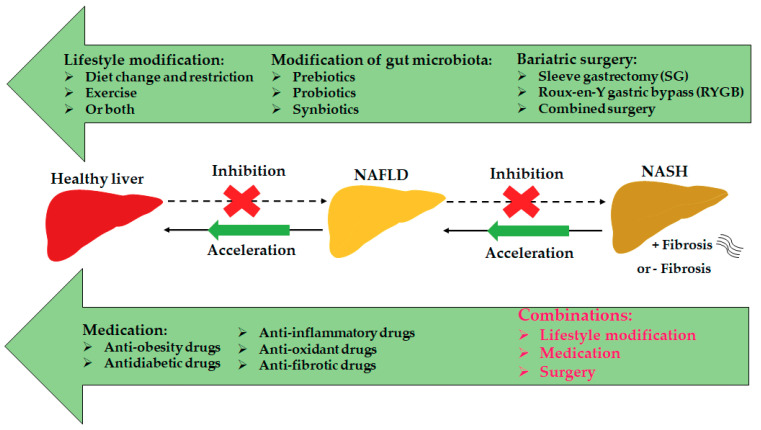
Treatment options for NAFLD or NASH. The currently effective treatment options for NAFLD/NASH include lifestyle modification, bariatric surgery, and medicines. In addition, modification of gut microbiota by prebiotics, probiotics, or synbiotics shows promising effects.

In addition, there are some other targets for NAFLD and NASH treatments, such as G protein-coupled receptors (*GPCRs*) [142], estrogen-related receptor alpha (*ERRα*) [143], bone morphogenetic proteins (*BMPs*) [144], and *KLFs* [24,145]. The treatment options for NAFLD/NASH are summarized in the graphic picture (Figure 2). Overall, regulating liver and gut metabolism, liver inflammation, fibrosis, and cell death can effectively prevent the development of NAFLD and NASH. 

## 5. Clinical Trials

This review summarizes the completed clinical trials targeting the improvement of NAFLD and NASH (Table 3). The data were collected from the website https://clinicaltrials.gov (accessed on 20 June 2021) with the keywords NAFLD, NASH, and treatment. The testing candidates may not provide the prospective effect as shown in preclinical studies. For example, a phase 2 clinical trial showed that *GLP-1* receptor agonist semaglutide can improve NASH resolution without worsening liver fibrosis compared to placebo, but semaglutide did not significantly ameliorate liver fibrosis in NASH patients [146]. 

This review summarizes the completed clinical trials targeting the improvement of NAFLD and NASH (Table 3). The data were collected from the website https://clinicaltrials.gov (accessed on 20 June 2021) with the keywords NAFLD, NASH, and treatment. The testing candidates may not provide the prospective effect as shown in preclinical studies. For example, a phase 2 clinical trial showed that *GLP-1* receptor agonist semaglutide can improve NASH resolution without worsening liver fibrosis compared to placebo, but semaglutide did not significantly ameliorate liver fibrosis in NASH patients [146]. 

## 6. Conclusions

The incidence of NAFLD and NASH is increasing currently, which is positively associated with the prevalence of obesity and diabetes. NAFLD and NASH are the major increasing factors that contribute to the progression of HCC, the primary liver cancer. However, there is no currently approved treatment for NAFLD and NASH. New non-invasive diagnostic markers such miRNAs have been evaluated for future diagnosis of NAFLD. Early diagnosis of the progression of NAFLD to liver fibrosis, cirrhosis, or HCC is critically important due to irreversible or difficulty to reverse of server liver disease. Some key molecules such as *PPARs*, *GLP-1*, miRNAs, and *KLFs* are potential targets for the treatment of metabolic diseases including NAFLD and NASH. Preclinical studies and clinical trials have been processed to evaluate potential treatment options for NAFLD and NASH, including synbiotics, pan-caspase inhibitors, *CCR2*/*5* antagonists, *FXR* agonists, and so on. A combined treatment such as combined medical treatment and physical activity could reduce the treatment time and improve the outcome. 

## Figures and Tables

**Table 1:** The miRNAs-mediated function and therapy in NAFLD.

miRNAs	Target	Function	Reference
*miR-21*	*PPARα*	In a diet-induced NASH model, *miR-21* ablation ameliorated the progression of hepatic steatosis, apoptosis, and fibrosis via inhibiting the expression of *PPARα*.	[57]
*miR-29a*	*HMGCR*	Overexpression of *miR-29a* in steatosis hepatic SMMC-7721 cells significantly reduced the accumulation of free cholesterol and the expression of 3-hydroxy-3-methylglutaryl coenzyme A reductase (*HMGCR*), a rate-limiting enzyme of cholesterol synthesis in the liver. Furthermore, the expression of *miR-29a* was inversely correlated with *HMGCR* expression in MCD-fed mice and two steatosis hepatic cell models (SMMC-7721 and HL-7702 cells), indicating that *miR-29a* can be utilized as a potential therapeutic target for the treatment of NAFLD.	[58]
*miR-34a*	*PPARα*	Inhibition of *miR-34a* expression suppressed lipid accumulation and improved the degree of steatosis, ameliorating the development of NAFLD by targeting *PPARα*.	[59]
*miR-122*	*Sirt1*	Knockdown of *miR-122* effectively decreased excessive lipid production and suppressed the expression of lipogenic genes in FFA-treated HepG2 and Huh-7 cells via upregulating *Sirt1* by binding to its 3’-untranslated region (UTR). In addition, *miR-122* knockdown activated the liver Kinase B1 (*LKB1*)/*AMPK* signaling pathway.	[60]
*miR-146a-5p*	*ROCK1*	It has been reported that nuclear enriched abundant transcript 1 (*NEAT1*) was significantly upregulated in the NAFLD model. *NEAT1* regulates the expression of *miR-146a-5p* that targets *ROCK1* (rho-associated, coiled-coil-containing protein kinase 1), which further affects the *AMPK*/*SREBP* pathway.	[61]
*miR-181a*	*PPARα*	Inhibition of *miR-181a* expression resulted in the upregulation of *PPARα* signaling pathway and inhibited palmitic acid (PA)-induced lipid accumulation in hepatocytes. The upregulation of *miR-181a* showed a reverse effect in hepatocyte lipid accumulation. Meanwhile, upregulating *PPARα* abrogated *miR-181a* mimics-induced lipid accumulation in hepatocytes. This study suggests that the downregulation of *miR-181a* may improve lipid metabolism in NAFLD.	[62]
*miR-192-5p*	*SCD-1*	In PA-treated Huh7 cells, overexpression of *miR-192-5p* significantly reduced lipid accumulation, which was abrogated by stearoyl-CoA desaturase 1 (*SCD-1*) siRNA. Transfection of *miR-192-5p* mimic and inhibitor in Huh7 cells dramatically repressed and promoted *SCD-1* protein expression, respectively.	[63]
*miR-205*	*NEU1*	*MiR-205* expression was inversely correlated with neuraminidase 1 (*NEU1*) expression in both HFD-fed mice and oleic acid (OA)-treated HepG2 and PH cells. In HFD-fed mice, overexpression of *miR-205* resulted in a decrease in body weight, liver weight and triglyceride, and lipid accumulation. The in vitro study indicated that overexpression of *miR-205* ameliorated lipid accumulation in OA-induced HepG2 and PH cells by targeting *NEU1*, identified by the TargetScan analysis and Luciferase assay. Knockdown of *NEU1* reduced lipid accumulation in vivo, suggesting that *miR-205* might be a therapeutic target for NAFLD.	[64]
*miR-873-5p*	*GNMT*	In hepatocytes of a preclinical murine NASH model, *miR-873-5p* controlled the enzyme glycine N-methyltransferase (*GNMT*) expression, which mediates mitochondrial functionality. Upregulation of *miR-873-5p* was also shown in the liver of NAFLD/NASH patients, correlating with hepatic *GNMT* depletion. Treatment with anti-*miR-873-5p* resolved lipid accumulation, inflammation, and fibrosis by enhancing fatty acid β-oxidation in the mitochondria, suggesting that *miR-873-5p* inhibitor emerges as a potential treatment for NASH.	[65]

**Table 2:** Serum markers and score system for NAFLD and NASH diagnosis.

Score/Marker	Test Components	Diagnosis	References
TG/HDL-C ratio	Triglycerides to high-density lipoprotein cholesterol ratio (TG/HDL-C)	Presence of NAFLD	[71,72]
Biglycan (*BGN*)	The cutoff value of 189.58 pg/mL of serum *BGN* with the best sensitivity (93.55%) and specificity (87.18%)	Fibrosis stage of NASH	[73,74]
NAFLD ridge score	ALT, HDL-C, TG, haemoglobin A1c, white blood cell count, the presence of hypertension	Presence of NAFLD	[75]
Hepatic steatosis index (HSI)	8× (ALT/AST ratio) + BMI (+2, if female; +2, if diabetes mellitus)	Presence of NAFLD	[76]
BARD score	BMI, AST/ALT ratio, diabetes mellitus	Presence of NAFLD	[77,78]
FIB-4 score	Age, platelet count, ALT, AST	Presence of NAFLD or fibrosis stage of NASH	[79,80,81]
NAFLD fibrosis score	Age, hyperglycemia, body mass index, platelet count, albumin, and AST/ALT ratio	Presence of fibrosis in NAFLD	[80,82]
Fatty Liver Index	BMI, waist circumference, triglycerides, and γ-glutamyltransferase	Presence of NAFLD	[83,84,85]
AUROC	Waist circumference, ALT, HbA1c, and HOMA-IR	Presence of NAFLD	[86]

Abbreviations: ALT, alanine aminotransferase; AST, aspartate aminotransferase; AUROC, area under the receiver operating characteristic curve; BMI, body mass index; FIB-4, fibrosis-4 score; HbA1c, hemoglobin A1c; HOMA-IR, homeostasis model assessment for insulin resistance; TG, triglyceride.

**Table 3:** The completed clinical trials for NAFLD/NASH treatment.

Liver Disease	Treatment	Target	Trials	References
NAFLD NASH	MSDC-0602K	Two higher doses of MSDC-0602K (125 mg and 250 mg), a second-generation thiazolidinedione (TZD) significantly reduced the levels of glucose, glycated hemoglobin, insulin, liver enzymes, and NAS compared to placebo.	NCT02784444	[147]
NAFLD	Volixibat	Treatment with volixibat for 12 days, a selective inhibitor of the apical sodium-dependent bile acid transporter, can inhibit bile acid reabsorption in overweight and obese adults.	NCT02287779	[148]
NASH	Cilofexor (GS-9674)	Therapy with Cilofexor for 24 weeks, a nonsteroidal agonist of *FXR*, was well-tolerated and provided significant reductions in hepatic steatosis, liver biochemistry, and serum bile acids in patients with NASH.	NCT02854605	[149]
NASH	Vitamin E	Vitamin E can decrease serum ALT levels and NAS score, but not liver fibrosis.	NCT00063622	[150]
NAFLD	Vitamin E	Treatment with vitamin E (α-tocopherol, αT) improved liver injury and steatosis.	NCT01792115	[151]
NASH	Firsocostat (GS-0976)	Treatment with GS-0976 for 12 weeks dramatically decreased serum level of ALT, liver de novo lipogenesis (DNL), steatosis, and stiffness.	NCT02856555	[152,153]
NASH	Pegbelfermin (BMS-986036)	Administration of Pegbelfermin for 16 weeks, a PEGylated human fibroblast growth factor 21 (*FGF21*) analogue, was generally well tolerated and significantly decreased liver fat.	NCT02413372	[154]
NAFLD	Obeticholic acid (OCA)	Treatment with *FXR* agonist OCA for 6 weeks improved insulin sensitivity and decreased markers of liver inflammation and fibrosis in patients with T2DM and NAFLD.	NCT00501592	[155]
NASH	Losartan	Treatment with losartan, an angiotensin II receptor blocker, improvement in alanine ALT, AST, and HOMA-IR compared to the placebo.	NCT01913470	[156]
NASH	Silymarin	Treatment with silymarin, an extract of milk thistle, did not significantly improve NAFLD Activity Score (NAS) and liver fibrosis.	NCT00680407	[157]
NASH	Metformin	Forty-eight weeks of metformin (2000 mg/day) therapy improved NASH activity index and ALT levels, and reduced bodyweight.	NCT00063232	[158]
NASH	Cenicriviroc (CVC)	Therapy with Cenicriviroc, *CCR2* and *CCR5* dual antagonist, showed an antifibrotic effect without impacting steatohepatitis at year 1 in responders, which was maintained in year 2 with greater effect in advanced fibrosis.	NCT02217475	[159]
NASH	Pentoxifylline (PTX)	Pentoxifylline, a competitive nonselective phosphodiesterase inhibitor, can improve liver steatosis and AST, ALT in patients with NASH compared to the baseline.	NCT00267670	[160]
NAFLD	Low free sugar diet	Eight weeks of use of a low-free sugar diet in adolescent boys with NAFLD resulted in significant improvement in hepatic steatosis compared to the usual diet.	NCT02513121	[161]
NAFLD	Synbiotics	Administration of a synbiotic combination of probiotic and prebiotic for one year changed gut microbiota but did not reduce liver fat content or markers of liver fibrosis.	NCT01680640	[112]
NAFLD	Emricasan	Treatment with Emricasan, a pan-caspase inhibitor, caused a reduction of ALT and cleaved cytokeratin-18, full-length cytokeratin-18, and caspase 3/7 in patients with NAFLD at day 7 and day 28 post-treatment.	NCT02077374	[162]

## Data Availability

All the data supporting reported results can be found in the paper.
